# Glycolysis: an emerging regulator of osteoarthritis

**DOI:** 10.3389/fimmu.2023.1327852

**Published:** 2024-01-09

**Authors:** Dingming Jiang, Jianan Guo, Yingquan Liu, Wenxin Li, Dezhao Lu

**Affiliations:** ^1^School of Life Sciences, Zhejiang Chinese Medical University, Hangzhou, China; ^2^The Second Affiliated Hospital of Zhejiang Chinese Medical University, Hangzhou, China; ^3^Hangzhou Linping District Nanyuan Street Community Health Center, Hangzhou, China

**Keywords:** glycolysis, osteoarthritis, immunometabolic reprogramming, inflammation, metabolism

## Abstract

Osteoarthritis (OA) has been a leading cause of disability in the elderly and there remains a lack of effective therapeutic approaches as the mechanisms of pathogenesis and progression have yet to be elucidated. As OA progresses, cellular metabolic profiles and energy production are altered, and emerging metabolic reprogramming highlights the importance of specific metabolic pathways in disease progression. As a crucial part of glucose metabolism, glycolysis bridges metabolic and inflammatory dysfunctions. Moreover, the glycolytic pathway is involved in different areas of metabolism and inflammation, and is associated with a variety of transcription factors. To date, it has not been fully elucidated whether the changes in the glycolytic pathway and its associated key enzymes are associated with the onset or progression of OA. This review summarizes the important role of glycolysis in mediating cellular metabolic reprogramming in OA and its role in inducing tissue inflammation and injury, with the aim of providing further insights into its pathological functions and proposing new targets for the treatment of OA.

## 1 Introduction

According to relevant statistics, osteoarthritis (OA) is the most common joint disease in humans, with at least 500 million people worldwide suffering from osteoarthritis, and its prevalence is on the rise as a result of a number of risk factors (e.g., metabolic syndrome, aging) (1). Patients with osteoarthritis are a large proportion of the elderly population and it is a major threat to the health of the elderly, causing pain and disability to the individual (2). Together with its socioeconomic costs, the prevalence of OA accounts for 1% to 2.5% of the gross domestic product of developed countries and these are expected to increase as life expectancy increases worldwide (3). Modern medicine has generally believed that OA is caused by genetics, age, dietary factor, bone density, ligament damage, poor alignment of knee joints, metabolism, occupation, etc. (4). There is still uncertainty about the etiopathogenesis of OA, but it has been suggested that it may be intricately related to inflammatory, metabolic, and mechanical processes (5).

More and more studies believe that OA is a local inflammatory joint disease, and synovitis is an important early pathological change (6). Inflammation promotes the progression of OA and exacerbates its clinical symptoms (e.g., pain, and swelling) (7). Recently, the main treatment options for OA include non-drugs (e.g., exercise, self-management), drugs (e.g., NSAIDs, opioids), and surgery (e.g., arthroscopy, joint replacement). However, there has been no significant progress in the delay and treatment of OA by drug therapy in recent decades, and the side effects caused by drugs are not universally applicable to some patients. As for surgery-related treatments, they may often be short-lived or have potentially dangerous complications (8). Therefore, an in-depth understanding of the pathogenesis of OA and seeking new therapeutic targets are helpful to suggest new means of treatment of OA and improve the quality of life of OA patients.

It is now being found that metabolic disturbances are strongly associated with OA. In terms of macro direction, OA may be closely related to metabolic diseases, such as Type 2 diabetes mellitus, cardiovascular diseases, and metabolic syndrome, because there is evidence that the metabolic pathways and factors of these metabolic diseases can have a direct impact on the joints (9, 10). Also, some studies have suggested that OA is the fifth component of metabolic syndrome (11). Besides, obesity- and diabetes-related inflammation and regulation of gut microbes have been linked to OA severity in some mouse models (10). Microscopically, the energy metabolism of the cells experiencing OA undergoes a dramatic shift. Typically, the energy metabolism generates energy from nutrients required to maintain basic cellular homeostasis, precise cellular activity, and normal function. There are a series of interrelated pathways involved in metabolism that function either aerobically or anaerobically, including glycolysis, the tricarboxylic acid (TCA) cycle, the pentose phosphate pathway, fatty acid oxidation, fatty acid synthesis, and amino acid metabolism. The changes in metabolism are the result of dysregulation of energy production, biomolecule synthesis (amino acids, nucleotides, fatty acids, lipids), failure of mitochondrial and reactive oxygen species (ROS) regulation, or energy-sensing signaling pathways (e.g., mammalian targets). As mentioned previously, chondrocytes obtain energy primarily from glycolysis, and in general, in healthy joints, they are in metabolic equilibrium. However, in osteoarthritis joints or inflammatory environments, the chondrocytes undergo metabolic changes (12). Impaired glycolytic metabolism can lead to hypertrophy of chondrocytes and degradation of extracellular matrix, which promotes the development of osteoarthritis (13). As a result, there is now an emerging thought that metabolic therapies targeting glycolysis-related metabolism could be used to treat OA. This paper will address four main questions: і) How much do the abnormalities of cellular glycolytic metabolism contribute to the development of OA? ii) What are the potential signaling pathways that control glycolytic homeostasis during OA? iii) Any relationship between glycolysis and programmed cell death during OA? iv) A review of the treatment options for OA is presented, as well as the future directions for the study of glycolysis in OA.

## 2 Osteoarthritis

The hallmark of OA is pathological changes in the joint structure, including cartilage degeneration, synovial inflammation, and subchondral sclerosis with osteophyte formation (14). Articular cartilage is a reduced cellular, avascular, neurogenic, and lymphoid tissue with limited oxygen and glucose availability (15). As the primary controllers of cartilage tissue metabolism, chondrocytes are the only cells present in articular cartilage (16). Articular hyaline cartilage consists of chondrocytes within the extracellular matrix (ECM), which is generated and maintained by chondrocytes (17). Compared to hyaline cartilage, the subchondral bone, synovium, and joint capsule have a direct vascular supply. It is lined with synoviocytes of type A and type B synoviocytes (18). A-type synoviocytes or macrophages act primarily as scavenger cells. B-type synoviocytes or fibroblast-like cells are responsible for the production of synovial fluid, which consists of hyaluronic acid and lubricin. Also, hyaluronic acid acts as a lubricant and balances with the hyaluronic acid in the ECM (19). Inflammation of the synovium, massive destruction of articular cartilage, and persistent joint destruction accompanied by severe joint pain in the later stadium are the most predominant pathologic features of OA (20). The specific pathogenesis of OA is well reviewed in Martel-Pelletier et al. (14) and will not be repeated here; this article will focus on the novel pathogenesis of OA involving glycolysis-related pathogenesis and the exploration of potential therapeutic options.

## 3 The role of glycolysis in osteoarthritis

**Figure 1:**
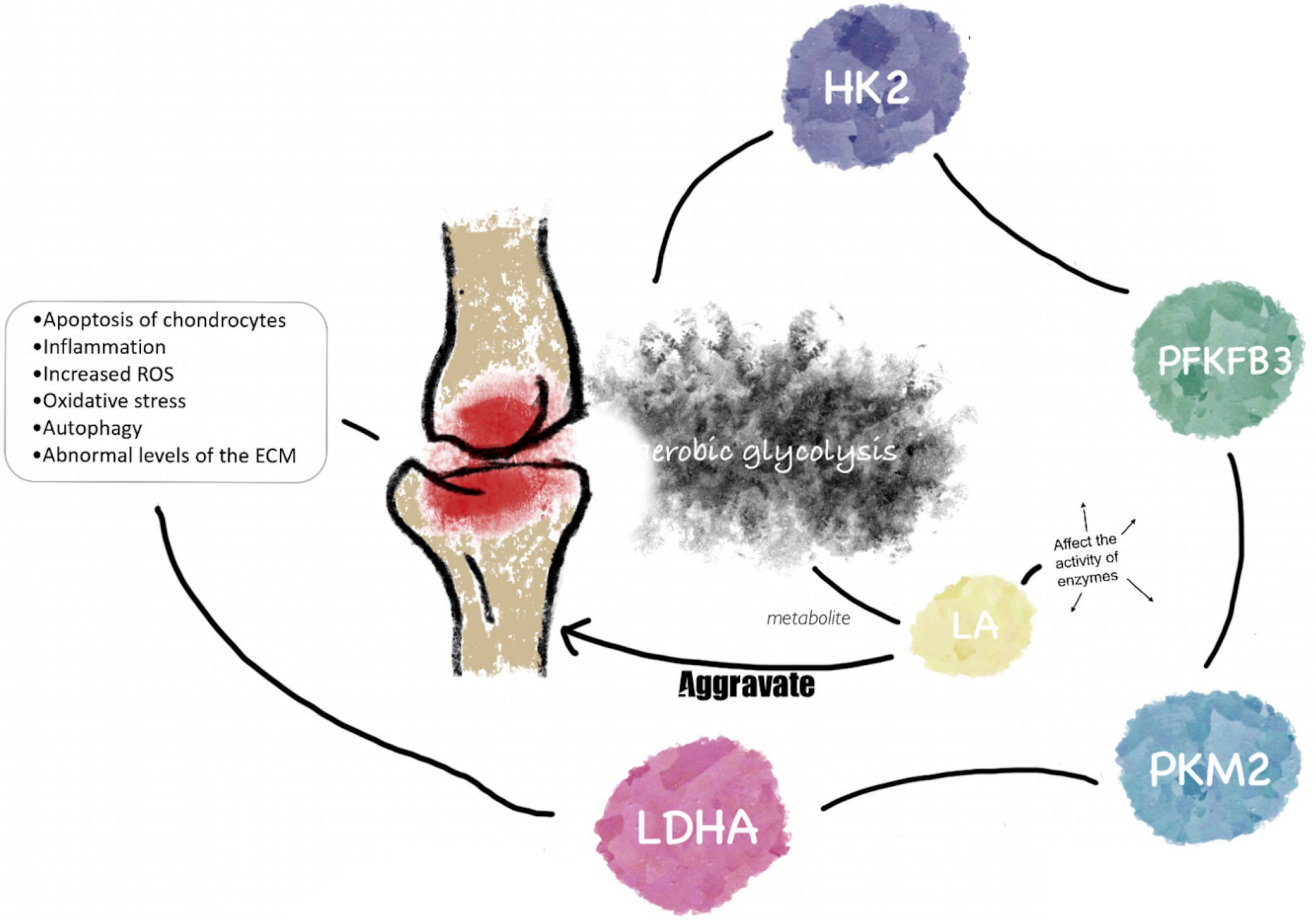
The link between glycolysis and osteoarthritis. HK2, PFKFB3, PKM2, and LDHA are important rater enzymes and kinases in aerobic glycolysis. They play potentially important roles in the pathological processes of OA, such as inflammation, apoptosis, oxidative stress, etc. LA, as an important metabolite of aerobic glycolysis, aggravates the progression of OA by regulating the activity of key enzymes of aerobic glycolysis in the pathogenesis of OA.

**Figure 2:**
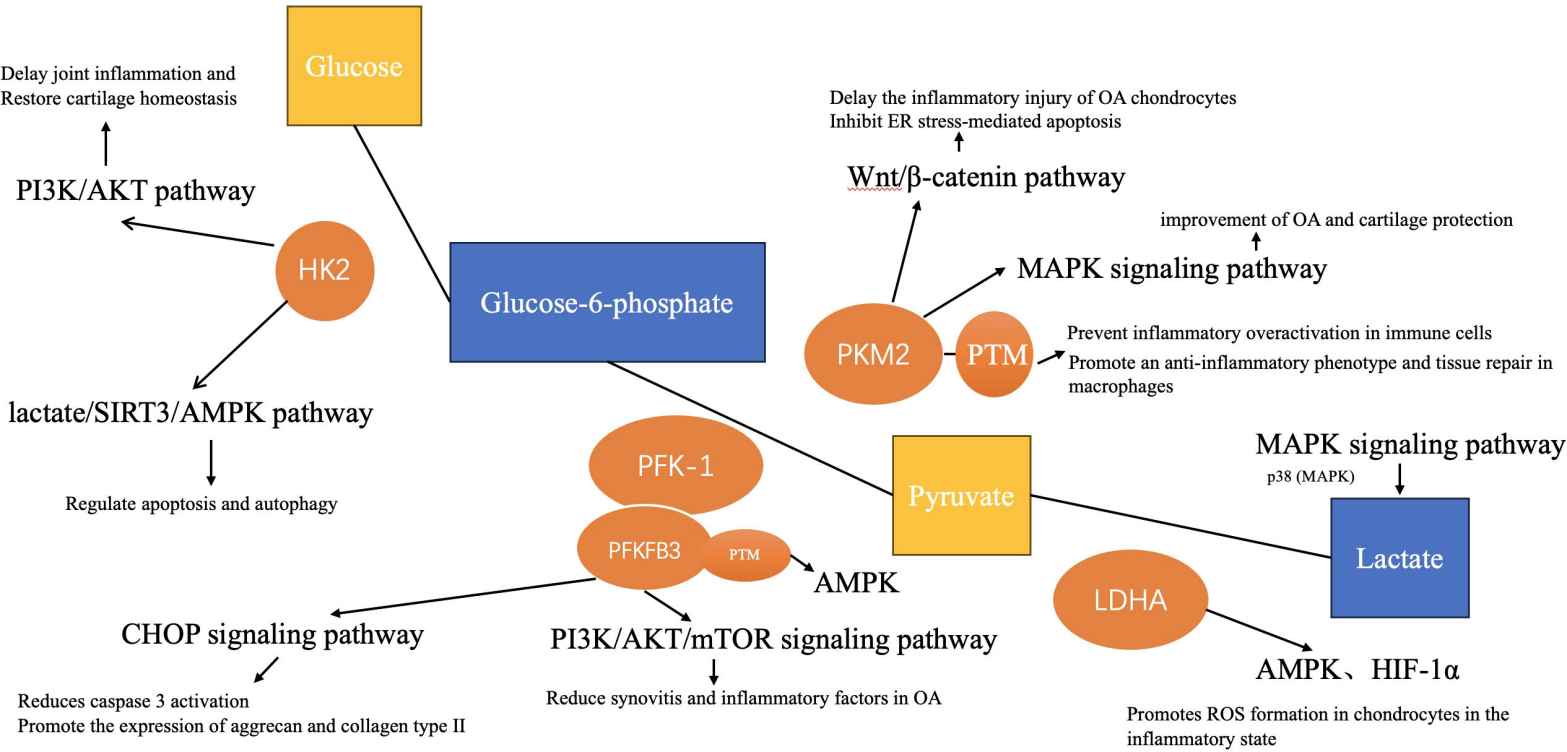
The link between glycolysis and osteoarthritis. The role of these enzymes and aerobic glycolysis in the pathological process of OA is related to some pathways, such as PI3K/AKT, HIF-1α, MAPK, etc.

A new perspective on the pathogenesis of OA has been found to be the changes in grape metabolism (including glycolysis, pentose phosphate pathway, and tricarboxylic acid cycle) in recent years. Several research has found that anaerobic and aerobic glycolysis (Warburg effect) coexist in chondrocytes of normal articular cartilage (9, 21). Moreover, there is growing evidence that the Warburg effect (i.e., metabolic switch from oxidative phosphorylation to glycolysis) is also a characteristic of the development of some arthritic diseases (22). Warburg effect means that under the condition of sufficient oxygen, glycolytic metabolism is still needed to provide sufficient energy for cell proliferation. This process involves many enzymes, such as pyruvate kinase M2 (PKM2), lactate dehydrogenase A (LDHA), hexokinase2 (HK2), and 6-phosphofructo-2-kinase/fructose-2,6-biphosphatase 3 (PFKFB3), and related studies have shown that the increase of these enzymes in OA chondrocytes can promote the occurrence of inflammatory reaction, apoptosis of chondrocytes, and other pathological changes (23). A significant relationship between the progression of OA and glycolysis has been further demonstrated, as levels of abnormal products of glycolysis (i.e., lactate) have been found to be significantly elevated in synovial fluid from patients with OA (24). Moreover, there is evidence that the pathological process of joints in OA (i.e., cartilage damage) is closely related to the disturbance of metabolic balance, including synthesis and breakdown (9). Chondrocytes undergo glycolysis-associated metabolic switching after exposure to IL-1β or TNF in osteoarthritis, and this phenomenon contributes to chondrocyte injury during which the link between further inflammation and metabolic dysregulation may be exacerbated (25). Increasingly, the role of glycolysis for OA chondrocyte function is also being focused on, for example, it was recently shown that the activator protein 1 (AP-1) transcription factor component, c-Fos, regulates chondrocyte cellular bioenergetics by balancing pyruvate fluxes between anaerobic glycolysis and the tricarboxylic acid cycle in response to OA signaling, controlling chondrogenic integrity in osteoarthritis (26). Additionally, recent studies also have shown that abnormal inflammation-mediated glycolytic metabolism in subchondral bone induces osteoarthritis. Intervention in the glycolytic process has appeared to be an accurate and feasible approach to the prevention and treatment of OA (27). In this section, we explore the contribution of key metabolic enzymes and metabolites of the glycolytic metabolic pathway to OA. Besides, glycolysis being a precisely regulated system in the cellular metabolic network, it is held in check by many regulatory networks. Here we also explore the role of several classical signaling pathways and protein Post-translational modification systems in the regulation of glycolysis in OA. The contribution of the glycolytic metabolic pathway to OA is summarized in **Figures 1**, **2**.

### 3.1 PKM2 and OA

An important regulator of glycolysis is pyruvate kinase (PK), which is an important limiting enzyme in glycolysis (28). And PKM2 is one of the mammalian pyruvate kinase isoforms. It is mainly expressed in proliferating cells, such as tumor cells and activated immune cells, when there is pathological cell conversion (29). There are two forms of PKM2, a dimer with low pyruvate kinase activity and a tetramer with high pyruvate kinase activity (30). These two conformations of PKM2 can be interconverted, as in the case of de/phosphorylated modifications, de/acetylated modifications, and so on (31). This conformational switch in PKM2 plays a key switch in glycolysis catalyzing the breakdown of glycolytic intermediates and the eventual generation of ATP, and is a key metabolic link in the process of the Warburg effect (aerobic glycolysis) (32). On the other hand, PKM2 can be used as a molecular integrator for metabolic disorders and inflammatory reactions in some diseases, and the reason is that it promotes the production of interleukin (IL)-6 and IL-1β and phosphorylates the transcription factor STAT3 as a protein kinase (33). To be specific, the dimerization of PKM2 induces its localization to the nucleus and binds to the promoter of downstream target genes such as HIF-1α, promoting the expression of hypoxia-inducible factor (HIF-1α), which activates inflammation-related target genes (IL-1β) and glycolysis-related target genes (e.g., lactate dehydrogenase (LDH), the glucose transporter protein GLUT-1, and the pyruvate dehydrogenase kinase), thereby further promoting the sustained overexcitation of glycolysis, the accumulation of lactate, and the amplification of inflammation in the cell, creating a positive feedback cascade (34, 35). Changes in such processes have been demonstrated in many diseases involving inflammation, and this phenomenon seems to have been recently recognized in OA as well (36).

Synovitis (an inflammatory reaction) plays an important role in the pathogenesis of OA, as studies have shown that 89% of patients with OA have periostitis, and many inflammatory mediators can be found in the synovitis and synovial fluid of patients with OA (20, 37). PKM2 targeting inflammation to alleviate rheumatoid arthritis has been demonstrated, and its role in regulating chondrocyte inflammation in OA has been further identified (38). In addition to an inflammatory reaction, the pathogenesis of OA also includes the degeneration of articular cartilage, and chondrocytes, as the only articular chondrocytes, play an important role in this process. The main energy source of OA is glycolysis because it has been shown that the ATP level of OA chondrocytes changes after treatment with 2-DG (glycolysis inhibitor), among which PKM2 is found to be involved in the regulation of ATP production and has a prominent function in glycolysis. OA cartilage also relies on PKM2 for collagen matrix production and chondrocyte energy regulation. A number of studies have shown that PKM2 inhibits proliferation and promotes apoptosis in OA chondrocytes. PKM2 expression in OA chondrocytes and its catalytic effect promote cartilage degeneration microenvironments (i.e. acidic microenvironments) (12). Additionally, previous studies have observed that the expression of PKM2 in OA chondrocytes is significantly higher than that in healthy human chondrocytes. The pathological process of increased chondrocyte loss has been demonstrated by apoptosis in degenerative diseases, such as OA. In these studies, it was also found that attenuation and loss of PKM2 could inhibit chondrocyte apoptosis and improve cell viability (12, 38).

Endoplasmic reticulum (ER) stress has been increasingly demonstrated to be a key etiology leading to the development of OA pathology (i.e., increased chondrocyte apoptosis and inflammatory cytokines) and disease progression. In a rat model, PKM2 knockdown significantly inhibited the expression levels of ER stress-related indicators (GRP78, CHOP, and C‐caspase‐12). PKM2 also decreased the inflammatory mediators produced by IL-1β stimulation accumulation (tumor necrosis factor-α and IL-6), and downregulated the expression of inducible nitric oxide synthase (iNOS) and cyclooxygenase‐2 (COX-2) induced by IL-1β. What’s more, the ablation of PKM2 inhibited IL-1β-induced the secretion of nitric oxide (NO) and prostaglandin E2 (PGE2), thereby alleviating chondrocyte apoptosis and inhibiting the inflammatory response of chondrocytes. This further indicates that the reduction of IL-1β-activated ER stress through PKM2 inhibition can be a potential target for the treatment of OA (38).

The Wnt/β‐catenin pathway activity is closely related to OA progression, and the Wnt/β‐catenin pathway can be triggered in IL‐1β exposed chondrocytes. In addition, Rspo2, a highly expressed positive regulator of bone metabolism in patients with OA, has been proposed to delay the inflammatory injury of OA chondrocytes by inactivating the Wnt/β‐catenin pathway and inhibiting Rspo2 (39). The involvement of PKM2 in the regulation of the Wnt/β‐catenin signaling via inhibition of IL‐1β‐ induced chondrocytes and silencing of Rspo2 expression reduction was confirmed by a previous study. These findings together suggest that PKM2 elimination can not only deal with the inflammatory injury of chondrocytes but also inhibit ER stress-mediated apoptosis by antagonizing the Rspo2/Wnt/β-catenin pathway (38).

Chondrocyte senescence is an important mechanism of osteoarthritis in the elderly population and is characterized by decreased expression of extracellular matrix proteins. It has been found that PMK2 regulates chondrocyte senescence, and the silencing of PKM2 decreases chondrocyte senescence. Furthermore, the silencing of PKM2 decreases IL-1β-stimulated elevated protein expression of matrix metallopeptidase (MMP)13 and PKM2 in primary chondrocytes and restores protein expression of type II collagen (COL2A1) (40).

In translated proteins, post-translational modification (PTM) occurs when amino acid side chains are covalently modified (41). Under physiological and pathological conditions, it regulates protein folding, activity, stability, localization, signal transduction, and binding (42). Its main forms include ubiquitination, phosphorylation, methylation, acetylation, glycosylation, glutathionylation, palmitoylation, lactylation and succinylation (43). PTM is gradually being studied in OA with respect to immune cell activation, signal regulation, immune response, and tumor metabolic reprogramming (44). The PTM of glycolytic intermediate related proteins affecting their function has been of great interest. Among the many glycolyzed proteins, the PTM of PKM2 is undoubtedly one of the most talked about, due to the fact that its conformational transition is influenced by the PTM (31). Tyrosine phosphorylation is the transfer of γ-phosphate to amino acids, which is a key mechanism for signaling in eukaryotic and prokaryotic cells. And it can occur at many cytoplasmic and nuclear residues, with serine, threonine and tyrosine being the most commonly phosphorylated residues in eukaryotic proteins. It was first reported that PKM2 can be phosphorylated at tyrosine 105 (45). In its physiological state, PKM2 is more active than when it is phosphorylated at tyrosine 105 (46). Based on this finding, it was subsequently demonstrated that phosphorylation at tyrosine residue 105 prevents PKM2 from promoting aerobic glycolysis and tumor growth as well as inflammatory overactivation in immune cells (45, 46). A significant conclusion drawn from several articles is that dephosphorylation of PKM2 promotes the Warburg effect in a variety of ways (47–49). In addition to phosphorylation, acetylation of PKM2 has been much studied. It usually involves the addition of an acetyl group from acetyl coenzyme A to an amino residue of the protein, and the reaction is mediated by acetyltransferases, which preferentially select the most commonly acetylated residues, such as lysine (50). The reverse reaction is accomplished by deacetylases, which primarily include SIRT1-7, which are typically zinc or NAD+ dependent. The acetylation of lysines 305 and 433 of PKM2 has been relatively well characterized. Where acetylation at lysine 305 was found to inhibit the enzymatic activity of PKM2 and promote its degradation through molecular chaperone-mediated autophagy (51), acetylation of PKM2 at lysine 433 promotes the translocation of PKM2 from the cytoplasm to the nucleus and promotes protein kinase activity (52). On the other hand, SIRT2, 3, 4, and 6 were all found to regulate the acetylation of PKM2 lysine site (51). The PTM of PKM2 also involves succinylation (53), SUMOylation (54), and nitrosylation (55). It is therefore noteworthy that PKM2 also undergoes lactylation. Lactylation is the transfer of L-Lactate to proteins to form protein lactylation (56). The lactylation of PKM2 differs from other PTMs in that it promotes not dimer formation but tetramer stabilization of PKM2 (57). Focusing on this, lactonylation of PKM2 promotes an anti-inflammatory phenotype and tissue repair in macrophages.

The MAPK signaling pathway has three branches, and its signal transduction is involved in cell apoptosis, cell proliferation, and cell differentiation (58). Some proinflammatory cytokines in OA, such as IL-1β, IL-6, and TNF-α, are regulated by MAPK. A pathway of MAPK signaling is closely associated with OA pathogenesis and influences OA progression (59, 60). It has been demonstrated that the levels of p38, JNK, and ERK are increased in OA and their phosphorylation is up-regulated in osteoarthritic cartilage (61–63). The mechanism has previously been suggested to be that increased phosphorylation of MAPK promotes osteoclast formation in cartilage and thus affects disease progression (64). Further study has confirmed that dihydroartemisinin can effectively inhibit the activation of osteoclasts through the MAPK signaling pathway (65). In addition, some studies have proposed that MAP kinase activated by MAPK phosphorylation can cause the upregulation of inflammatory factors in OA by activating and regulating other proteins, and the upregulation of inflammatory factors can further activate MAPK and cause positive feedback of inflammation in OA (60). Some studies have also proposed that the MAPK signaling pathway can regulate autophagy and ECM metabolic disorders in OA, thereby affecting the role of cartilage degradation in OA (66). The role of inflammatory factors and anti-inflammatory factors produced by M1/M2 macrophages in synovial membrane in OA has also been established by some studies, suggesting that macrophage polarization plays a regulatory role in OA (67, 68). Generally, M1 polarization promotes inflammation and tissue damage and while M2 polarization gives the ability to anti-inflammation and tissue repair. Furthermore, recent studies have shown that some drugs, such as kinsenosid and fargesin, can modify the number and function of M1 and M2 macrophages and have the ability to change the polarization transformation of M1 and M2 macrophages by modulating the signaling of the MAPK signaling pathway. Thus, it can reduce the expression of inflammatory factors in the synovium of OA and protect against the breakdown of OA chondrocytes and cartilage degeneration (69, 70). It has been found that the improvement of OA and cartilage protection can inhibit the aerobic glycolytic activity of OA by regulating some genes (including PKM2, an important glycolytic enzyme) through the MAPK signaling pathway and AKT signaling pathway by achyranthes bidentata extracts (71).

### 3.2 HK2 and OA

Hexokinase (HK) is a regulator of glucose metabolism and the first rate-limiting enzyme in aerobic glycolysis. In mammals, the HK family includes five isotypes (e.g., HK1, HK2, GCK, etc.), among which HK2 is the most efficient enzyme in promoting aerobic glycolysis among the HK isotypes (72, 73). The key role of HK2 in glucose metabolism lies in its unique kinetic properties. HK2 protein has a large molecular weight, and both its N-terminus and C-terminus have catalytic activity, and both are sensitive to the inhibitory effect of glucose-6-phosphate, making it highly affinity for glucose. Moreover, the predominant enzyme expressed in the musculoskeletal system is HK2 (23, 74). HK2 expression in tumor cells promotes conversion to aerobic glycolysis, which provides a large amount of energy and leads to excessive glucose uptake, as demonstrated by previous studies. Abnormal glucose metabolism has also been found in arthritis. OA is a pathological change thought to be due to chondrocyte hypoxia, mitochondrial dysfunction, and oxidative stress, and previous studies have demonstrated that HK2 plays an important role in chondrocyte-induced glucose metabolism reorganization in these pathological changes through its association with voltage-dependent anion channels. Besides, it has been found that HK2 plays a key role in regulating the abnormal metabolism of musculoskeletal cells under pathological conditions. HK2 involvement in different types of metabolic pathways through the mitochondria - ER has also been established (75). In addition to its role in aerobic glycolysis, HK2 also plays a role in the regulation of apoptosis (i.e., cell damage and cell viability after apoptosis). Moreover, HK2 also plays a direct or indirect role in the transcriptional activation and phosphorylation of certain signaling molecules in muscle and bone tissues (23). Through the above unique biological characteristics and functions of HK2, the possible role and mechanism of HK2 in OA are further discussed. The development of OA is closely related to chondrocyte metabolism and synthesis under hypoxic conditions, and HK2 is an important kinase in the pathological state of glycolysis (e.g., apoptosis). The level of HK2 protein in OA-FLS (fbroblast synovial -like cell lines) is closely related to tumor necrosis factor (TNF) and hypoxia, so the expression of HK2 in OA has been further confirmed.

Inflammation is the driving factor of OA, and NOD-like receptor protein 3 (NLRP3) expression level is significantly increased in the synovium of articular cartilage of OA patients. NLRP3 is involved in the pathogenesis of OA, and studies have also shown an important link between NLRP3 and glucose metabolism. In addition, it has been proposed that enhanced HK2 expression is positively correlated with the activation of NLRP3 inflammasome associated with inflammation (76). HK2 expression regulates the levels of proinflammatory cytokines (IL-6, IL-8) in OA (77). Transforming growth factor beta1 (TGF-β1) is a multifunctional cytokine that regulates cells (e.g., cell growth, cell differentiation, cell proliferation, etc.), not only participates in the regulation of glycolysis but also maintains and regulates the internal environment of articular cartilage. It has been demonstrated that TGF-β1 can increase HK2 expression in a time-dependent manner, thus conducting direct regulation. A previous cellular study has found that treatment of chondrocytes isolated from femoral condyles of patients with OA with TGF-β1, a cartilage protective factor and an important regulator of cartilage homeostasis, increased the expression of HK2 and glucose transporter 1(one of the key regulators of glycolysis), thereby stimulating enhanced aerobic glycolysis and increasing the production of abnormal metabolites (78). Therefore, the changes of HK2 in aerobic glycolysis in response to TGF-β1 provide important hints for promoting the changes in OA pathogenesis. The phosphoinositide 3-kinase/protein kinase B (PI3K/AKT) pathway can be used to regulate glucose metabolism and HK2 in glycolysis. Inhibition of AKT (a serine/threonine protein kinase) activation and blockade of the PI3K/AKT pathway can increase the activity of HK2 in aerobic glycolysis, thereby delaying joint inflammation and restoring cartilage homeostasis in OA cartilage. Therefore, the effect of HK2 on OA through the PI3K/AKT pathway further suggests that HK2 plays an important role in the pathogenesis of OA (79, 80). Tan et al. have found that HK2 could be affected by interfering with the lactate/SIRT3/AMPK pathway to regulate apoptosis and autophagy from aerobic glycolysis (81).

The expression of HIF-1α and HIF-2α in articular cartilage in OA is significantly higher than that in normal articular cartilage, and the expression of them in articular chondrocytes can be further induced to increase under pathological conditions (i.e., hypoxia) (82–84). HIF-1α activity is increased in OA chondrocytes and induces chondrocyte proliferation (82). Besides, HIF-1α expression affects articular cartilage degeneration in OA by affecting metabolic stress, oxidative stress, the levels of ROS and ECM gene, chondrocyte activity, and inflammatory response (85). Moreover, HIF-1α is essential in articular cartilage as a maintainer of ATP and a regulator of apoptotic factors (86, 87). Autophagy in OA is also regulated by HIF-1α through the mTOR pathway and AMPK activation (86). HIF-2α can be used to mediate SOX9 in OA to affect cartilage matrix synthesis, and induce the expression of some catabolic factors (including some matrix metalloproteinases, inflammatory factors, etc.) to affect the destruction and degradation of articular cartilage (88). The apoptosis level of chondrocytes in OA was in direct proportion to HIF-2α (89). Moreover, HIF-1α also plays an important role in the regulation of chondrocyte autophagy in OA (84, 90). The balance between HIF-1α and HIF-2α also plays a key role in the regulation of chondrocyte activity through the regulation of apoptosis and mitochondrial autophagy (84). HIF-1α and HIF-2α are also important regulators in aerobic glycolysis, affecting metabolic transport, transcription, and expression (91). HIF-1α stability can be maintained by glycolysis in both normoxic and hypoxic environments (92). The activation of the PI3K/AKT signaling pathway and the induction of mTOR is involved in the regulation of several glycolysis-related rate-limiting enzymes expression that is mainly dependent on HIF-1α activity and stability have also been proposed by previous studies (91, 92). It has been proposed that abnormal glycolytic metabolism improvement and self-repair in OA can up-regulate the expression of HIF-1α through the activation of Runt-related transcription factor 2 (RUNX2) (93). Furthermore, it has been demonstrated that glycolysis of chondrocytes can be regulated to protect cartilage by upregulation of HIF-1α in chondrocytes with icariin (94). The application of HIF-2α in cartilage glycolysis has also been further confirmed (95). Wang et al. have demonstrated the value of the NF-κB/HIF-2α signaling pathway in cartilage glycolysis and shown the intervention of anaerobic glycolysis by icariin to reduce induced cartilage degradation and inflammation (95). HIF-1α can control the mechanism of glycolysis by affecting the HK2 involved in catalyzing and rate-limiting glucose metabolism. The regulation of cytokines during OA inflammation by targeting HIF has been demonstrated by many studies, but there is still a lack of evidence for the targeted regulation of glucose metabolic pathways (especially aerobic glycolysis) by HIF in OA chondrocytes (96, 97).

### 3.3 LDHA and OA

Lactate dehydrogenase (LDH) is a tetrameric enzyme that catalyzes the forward and backward conversion of pyruvate to lactate in aerobic glycolysis, and it contains several isoenzymes, such as A1B3, A2B2, and A3B1, etc. The predominant form of LDH in skeletal muscle is LDHA (98). LDHA plays an important role in the Warburg effect, and its activity is positively correlated with aerobic glycolysis (99). At present, the application of LDHA in tumor tissues has been confirmed by some studies, and its reduction *in vivo* can delay tumorigenesis. Le, Anne et al. have proposed that this possible mechanism is hypoxia in tumor tissue, and the reduction of LDHA may affect cellular oxygen consumption and cell activity (98). Thus, it can be further extended to OA because inhibition of tumorigenesis affects oxidative stress production by reducing LDHA and this pathological mechanism is similar to that of OA.

Li, Hui Min, et al. have demonstrated that the positive staining of LDHA was significantly enhanced in temporomandibular joint OA (TMJOA) synovial tissues compared with normal synovial tissues (especially in the inflammatory synovial lining and thickened sublining cells) (24). One of the pathological mechanisms of OA is an inflammatory reaction, and previous studies have shown that OA chondrocytes switch to aerobic glycolysis in response to inflammation, and gene set analysis of chondrocytes treated with inflammatory cytokines, such as IL-1β, showed a marked increase in LDHA expression. In addition, inflammatory response genes in OA (e.g., IL-6 and matrix metalloprotease 13 (MMP13)) can be inhibited by LDHA inhibitors. Besides, several studies have confirmed that reactive oxygen species (ROS) is one of the important factors driving inflammatory reactions, such as OA. ROS can be good or bad molecules depending on their properties (e.g., size of production, duration, etc.). For example, ROS is signal transduction in normal physiological function, but it is a pathological risk when it is a mediator in the progression of OA disease. Furthermore, ROS are closely related to glycolysis, and LDHA, for example, plays a key role in their regulation and metabolism (100, 101). It has been proposed that ROS can affect OA articular chondrocytes through LDHA-mediated effects, and therefore reducing ROS formation function by inhibiting LDHA is an effective way to inhibit inflammation (100).

AMP-activated protein kinase (AMPK) activity plays a key role in chondrocytes in OA, which can affect the inflammatory response in OA and the development and progression of OA, and has been demonstrated to play an important role in the glycolysis of OA (102–104). Previous studies have also linked LDHA to OA by regulating AMPK function. The reduction and blockade of LDHA inhibit the secretion of abnormal glycolytic products (e.g., Lactic acid) and attenuate glycolytic disorders (thereby inhibiting the expression of inflammatory factors in OA) in synovial tissues OA. In addition, LDHA blockade also plays a role in the regulation of the OA microenvironment (i.e., hypoxia, acidity, high HIF-1α). Besides, previous research has suspected that the damage to cartilage and bone in OA may be related to the abnormality of LDHA during aerobic glycolysis. Therefore, LDHA inhibitors can be used as a way to delay the progression of OA and even reverse the pathological state of OA in future treatment (24).

### 3.4 PFKFB3 and OA

The involvement of 6-phosphofructo-1-kinase (PFK-1) as a regulator in catalyzing aerobic glycolysis is another important rate-limiting step. The affinity of PFK-1 for fructose-6-phosphate (F6P) is mediated by its potent activator (i.e., fructose 2,6-bisphosphate (F2,6P2)), which converts F6P to fructose-1,6-bisphosphate (F1,6P2). The PFK-2/FBPase (PFKFB) family has four isoenzymes with different properties, but together they control the cellular homeostatic concentrations of F2,6P2. As the highest kinase in the PFKFB family, PFKB3 has a particularly different gene coding composition. And the glycolytic pathway can be efficiently promoted through the function of two homologous subunits of PFKFB3 (105, 106). The regulation of PFKFB3 in the physiology and pathology of some tissues has been confirmed by current studies, such as its role in regulating cell expression and viability in tumor tissues, and its role in insulin and inflammatory response in adipose tissues. And recently, the role of PFKFB3 in OA has also been more and more elaborated. For example, it has been confirmed that OA cartilage explants transfected with AdPFKFB3 promoted aerobic glycolysis (i.e., increased production of ATP and lactate) in a previous experiment (11).

In studies related to cancer and aerobic glycolysis, the interaction between HIF-1α and PFKFB3 gene expression has been found to affect glycolysis progression and cell activity (107). However, the expression of HIF-1α is closely related to the apoptosis of chondrocytes in OA, and it also plays a certain role in the repair of glycolytic metabolism in OA (23, 84). In addition to mentioning the mechanism of ROS in OA, previous studies have shown that the decrease in ROS is related to the degradation of PFKFB3, further establishing the potential correlation between OA and PFKFB3 (105, 108).

In addition, the expression of PFKFB3 is also significantly increased in the pathological state of inflammation and hypoxia. In some cancer treatment studies, the PFKFB3 gene expression level has been found to interact with proinflammatory molecules (e.g., IL-6), thus this pathological mechanism similar to OA can be extended and deserves further study (105). Li et al. have described the changes in PFKFB3 levels in OA cartilage tissue and the influence of cytokines associated with the development of OA (e.g., TNF‐ α, IL‐1β) on PFKFB3 regulation. PFKFB3 (as an important enzyme in aerobic glycolysis) also plays an important role in ER stress in cartilage explants and chondrocytes of OA has been proposed (109). ER stress has been mentioned above as an important cause of OA. Recently, several studies have found changes in the expression of ER stress-related factors, such as PERK, ATF3, MMP13, p-eIF2α, and IRE1, in OA cartilage explants transfected with AdPFKFB3. Also, the expression of PFKFB3 was inversely proportional to the expression of these ER stress factors, which further confirmed the important regulatory role of PFKFB3 in OA. Besides, it has been established that PFKFE3 delays the development of OA by regulating the PI3K/Akt/C/EBP homologous protein (CHOP) signaling pathway. It has been reported that the expression of the CHOP signal plays a key role in improving the viability of OA chondrocytes. Related studies have found that the expression levels of P-Akt and CHOP in chondrocytes transfected with Adenovirus-packaged PFKFB3 cDNA (Ad-PFKFB3) are changed. Furthermore, PFKFB3 expression can affect the survival rate of OA chondrocytes by regulating the expression levels of P-Akt and CHOP, which can be restored by PI3K inhibitor. In addition, since the relationship between CHOP and PFKFB3 has been established, some studies have further confirmed the relationship between PFKFB3 and the progression of OA by finding that PFKFB3 can affect the expression of cleaved caspase 3, aggrecan, and type II collagen in OA chondrocytes (11).

The PTMs of PFKFB3 have also been brought to prominence recently. It was found that under energy deficiency, PFKFB3 is phosphorylated at residue S461 by AMP-activated kinase (AMPK), and that this phosphorylation increases the activity of PFKFB3, stimulating glycolysis and ATP production (110). AMPK is a protein kinase that acts as an energy sensor. In glucose metabolism, the activation of AMPK plays a role in affecting the activities and functions of rate-limiting enzymes and metabolic enzymes, so AMPK is closely related to changes in cellular ATP levels (111, 112). In addition, AMPK activation also affects oxidative stress and autophagy through several signaling pathways (e.g., NF-κB signaling pathways) (113). Previous studies have demonstrated dysfunction of AMPK activity in OA, and AMPK is essential for protecting normal chondrocyte homeostasis (9, 114). AMPK reactivity is decreased in articular cartilage due to biomechanical damage of articular cartilage and cellular aging factors, which positively affect the self-repair ability of articular cartilage (115). Besides, several drugs (e.g., quercetin and safflower yellow) that promote AMPK phosphorylation have been found to delay the progression of OA by reducing chondrocyte apoptosis, inhibiting inflammatory factors (e.g., TNF-α, IL-1β, and MMP-13), and modulating ER stress (116, 117). AMPK signaling also affects ROS, which is an important pathological factor in OA (118). In addition, AMPK dysfunction not only affects synovial inflammation in OA but also affects the balance of bone metabolism (119). Activation and inhibition of AMPK can affect the differentiation of osteoblasts and osteoclasts, thus acting as a regulatory mechanism of joint bone homeostasis (104, 120). It has been proposed that AMPK is an important negative regulator of aerobic glycolysis in tumor cells, and its activity can drive the Warburg effect and promote ATP production to inhibit cell proliferation. In the treatment of some diseases (e.g., lymphoma), it can be treated by targeting AMPK to participate in metabolic reprogramming (121). AMPK and glycolysis are closely interrelated, in which glycolysis is the key to AMPK activation, and AMPK can regulate the activation of PFKFB3 in aerobic glycolysis (122–124). Whereas PFKFB3 is acetylated at lysine 472 (K472), nuclear localization signaling (NLS) is reduced and accumulates PFKFB3 in the cytoplasm (123). The cytoplasmic accumulation of PFKFB3 promotes its phosphorylation by AMPK, leading to PFKFB3 activation and enhanced glycolysis. It would be worthwhile to further explore the PTM of more glycolysis-related proteins. Moreover, it has been demonstrated that p38 (MAPK) is involved in the proliferation of tumor cells and affects the signaling of aerobic glycolysis by affecting the activity and level of a PFKFB3 in aerobic glycolysis and glucose uptake.

The mechanistic target of rapamycin (mTOR) is a sensor and binding hub for cellular nutrition, including the regulation of cell growth and metabolism, because it consists of two protein complexes (mTORC1 and mTORC2) with distinct biological functions (125). In hypoxia, mTORC1 can regulate cell activity, and mTORC2 can activate AKT to induce glycolytic enzyme expression and thus affect cell function (126). Also, mTOR is a key factor in inhibiting autophagy because of the regulatory function of mTORC1 (127). Some studies have proposed that inhibition of autophagy can affect the progression of OA by causing chondrocyte apoptosis through mitochondrial dysfunction, so the knockdown of the mTOR signaling pathway can improve the balance between synthesis and degradation of chondrocyte extracellular matrix in osseous joints (128). In addition, the regulatory function of the mTOR signaling pathway also plays a certain role in the inflammatory response in OA. It has been suggested that the activity and production of proinflammatory cytokines (e.g., IL-1β) are affected by the inhibition of autophagy (129). Moreover, a previous study has demonstrated the increased expression of mTOR in peripheral blood monocytes of patients with OA (130). The PI3K/AKT signaling pathway is closely related to the homeostasis of the cartilage microenvironment, and it is significantly down-regulated in osteoarthritic chondrocytes. Moreover, the inhibition of the PI3K/AKT signaling pathway can be affected by an inflammatory response and oxidative stress. Studies have confirmed that chondrocyte apoptosis in OA is mainly regulated by PI3K/AKT signaling pathway. In addition, activated PI3K/AKT can effectively prevent chondrocyte degeneration and death (131). Inhibition of the PI3K/AKT/mTOR signaling pathway to reduce synovitis and inflammatory factors in OA has also been proposed (132). Furthermore, the influence of subchondral bone on the development of OA has also been recognized by more and more studies (133). Several studies believe that the structural instability of subchondral bone causes degeneration and thus subchondral sclerosis. However, relevant studies have found that the most important metabolic pathway of subchondral bone is the PI3K/AKT/mTOR signaling pathway (134). Among them, it has been confirmed that mTORC1 can affect the formation and structural changes of subchondral bone in OA (134). It has found that inhibition of the PI3K/AKT/mTOR signaling pathway blocks the process of aerobic glycolysis (e.g., decreased lactate production) in cells, and these studies speculate that it may regulate the expression of the PFKFB3 of aerobic glycolysis (135). Overall, the PTM of these proteins in glycolysis is important for glycolytic regulation, which is worth focusing on for therapeutic and mechanistic studies of OA.

### 3.5 Lactate (LA) and OA

Lactate is the most important metabolites of aerobic glycolysis. Some previous study has shown that the steady-state concentration level of lactate (i.e., the abnormal product of glycolysis) interacts with OA, and reducing lactate concentration dependence by inhibiting the glycolytic activity of OA can delay the progression of OA (24). Li et al. have found that dexamethasone used for the treatment of OA can affect the metabolic dysfunction of chondrocytes and affect the key genes of aerobic glycolysis, resulting in the accumulation of LA in chondrocytes and aggravating the progression of OA (136). Further, previous studies have shown that LA can regulate autophagy and apoptosis in OA by processing SIRT3 and AMPK treated with aerobic glycolytic rate-limiting enzymes (81). Relevant studies have suggested that the MAPK signaling pathway is an essential pathway for aerobic glycolysis in pancreatic cancer, and the expression of p38 (MAPK) in cells can affect the secretion of aerobic glycolysis products (including Lactate) (137, 138). Besides, lactate production can affect the activity and function of enzymes related to aerobic glycolysis, such as HK2, which are potential regulators of pathological metabolism and targeted therapeutic sites in OA, thus providing insights for further study of the interaction between OA and LA (81).

## 4 The regulated programmed cell death and glycolysis

**Figure 3:**
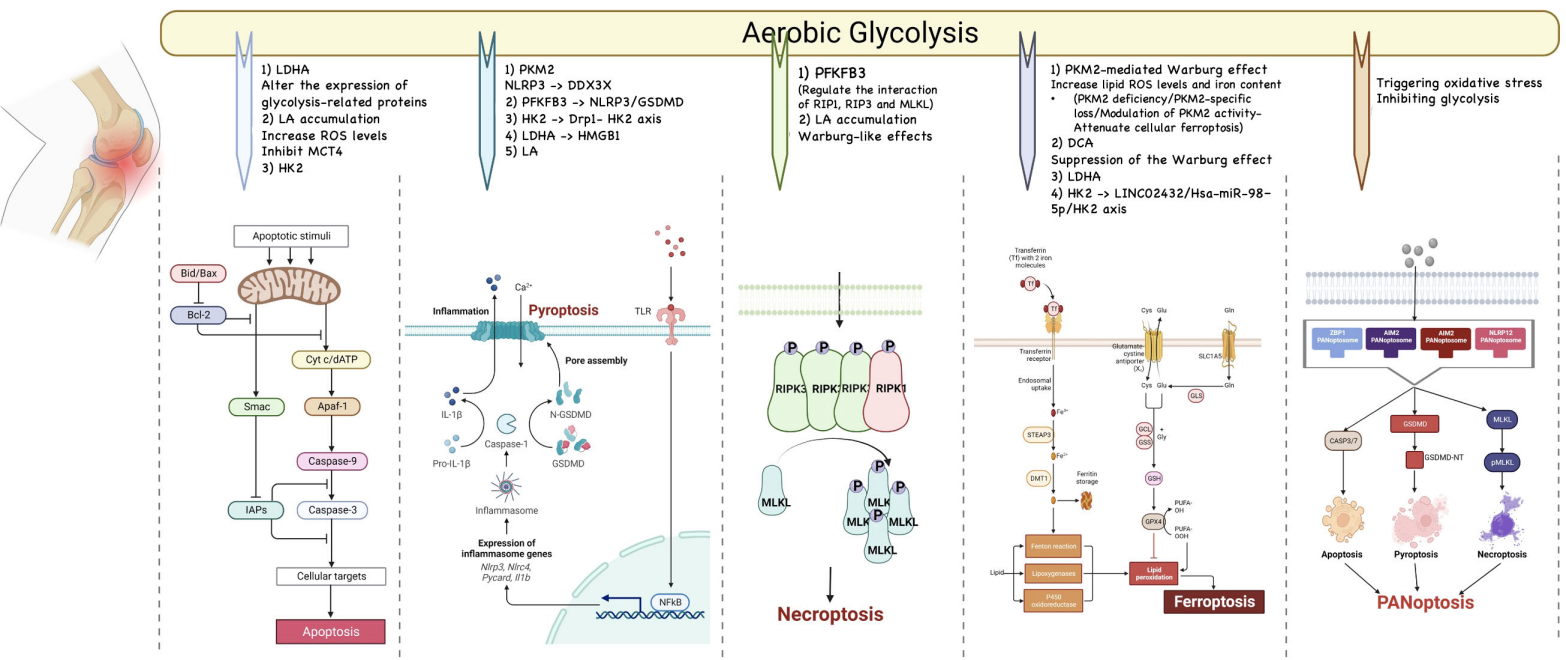
The relationship between glycolysis and programmed cell death. Many types of PCD have been identified, most of which, such as apoptosis, pyroptosis, PANoptosis, necroptosis, and ferroptosis could induce or exacerbate OA. Targeting PCDs may be a viable option for the treatment of OA. And several recent studies have revealed a link between glycolysis and PCDs.

Programmed cell death (PCD) is the mode of active cell death that is regulated by a variety of genes. All types of PCDs play an important role in organismal processes, and uncontrolled PCDs can lead to adverse consequences. Many types of PCD have been identified, most of which, such as apoptosis, pyroptosis, PANoptosis, necroptosis, ferroptosis, and cuproptosis could induce or exacerbate OA. Targeting PCDs may be a viable option for the treatment of OA. And several recent studies have revealed a link between glycolysis and PCDs. Therefore, we address the existing link between glycolysis and PCDs in this section and we open a discussion on targeting glycolysis to alleviate OA by attenuating PCDs. These links have been summarized in **Figure 3**.

### 4.1 Apoptosis and glycolysis

As early as 1972, the term “apoptosis” (a-po-toe-sis) was created by Kerr et al. to describe a morphologically distinct form of cell death (139). Apoptosis has been a widely studied form of PCD and it has been shown to play an important role in a variety of processes, including cell renewal, immune system development, hormone-dependent atrophy, embryonic development, and chemically induced cell death (140). In the pathological study of OA, apoptosis has been observed since early times and today chondrocyte apoptosis has been suggested to be an important factor in the pathogenesis of OA (141). Although, apoptosis removes damaged cells in an orderly and efficient manner, the dysregulation of this order can facilitate the development of OA (141). Caspase family proteins are regarded as both initiators and executors of apoptosis. To date, two types of caspases have been defined: caspases responsible for the initiation programme and caspases responsible for the effector/execution programme (142). Caspase-8 and -9 are treated as initiator caspases, while caspase 3 is an effector caspase. In addition, other caspases such as caspase-2, -10, and -11 were grouped into the initiator caspase category, while caspase-6 and -7 were placed in the effector caspase category. It is supported by research that activation of caspases can be achieved in two ways, intrinsic (or mitochondrial) and extrinsic (or death receptor), both of which can result in a common pathway or stage of execution (143). These potential mechanisms of apoptosis have an essential role to play in the pathogenesis of OA.

Several potential associations have been given, excessive oxidative stress, respiratory chain abnormalities and imbalances in mitochondrial dynamics leading to mitochondrial dysfunction, as well as the fact that both endogenous and exogenous NO can induce apoptosis through mitochondria-dependent mechanisms (144). The dysfunction of mitochondria can block mitochondrial respiration and the production of cytochrome C (Cyt C) and caspase-9 (145). On the other hand, the excessive levels of ROS can lead to the opening of the inner mitochondrial membrane containing a large conductance channel called PTP, resulting in the loss of the mitochondrial permeability transition pore (MMP), which causes the facilitation of the migration of cytochrome C (Cyt-C) from the mitochondrial matrix to the cytoplasm, which triggers apoptosis with the activation of caspases and the increase in the ratio of BAX/Bcl-2 (146). The increased burden of ROS is also concerned with chondrocytes having greater mtDNA damage, resulting in STING overexpression, which can accelerate apoptosis (147). Many non-coding RNAs, including long-stranded non-coding RNAs and cyclic RNAs, have also been shown to regulate chondrocyte apoptosis through different mechanisms (148–150).

Lots of studies have given the potential mechanism for the regulation of apoptosis by glycolysis. Previous studies have suggested that HK2 is involved in the apoptosis of hepatocellular carcinoma while affecting its glycolysis (151). Nonylphenol can reverse apoptosis and reduce intracellular ROS levels by inhibiting HIF-1α-induced aerobic glycolysis (152). Apoptosis and glycolysis of cancer cells have direct and close interaction in the process of tumor development (153). It is supported that the glycolytic pathway or some of its key proteins can regulate chondrocyte apoptosis in the course of OA. The LDHA inhibitor oxalate has been shown to reduce chondrocyte apoptosis in articular cartilage of the experimental osteoarthritis rat model by possibly altering the expression of glycolysis-related proteins (154). Dexamethasone has been found to increase ROS levels and inhibit monocarboxylate transporter 4 (MCT4), leading to apoptosis caused by lactate accumulation (136). Moreover, in the study of ABE intervention in osteoarthritis, it was found that Achyranthes bidentata (ABE) can regulate apoptosis to promote cell proliferation and target the glycolysis pathway, and protein kinase B has been shown to regulate both glycolysis and apoptosis of chondrocytes in the process that affects the function of chondrocytes (71). Although some studies have supported the association between the glycolytic pathway and chondrocyte apoptosis, the exact mechanism of how to regulate apoptosis by targeting the glycolytic pathway in OA seems to be unclear. Whether the enzymes and pathways involved in the process of glycolysis have more potential targets still needs to be further explored and studied.

### 4.2 Pyroptosis and glycolysis

Pyroptosis is a form of inflammatory cell death triggered by certain inflammatory vesicles, which leads to a massive extracellular release of inflammatory factors such as IL-18 and IL-1β, thereby inducing an inflammatory response (155, 156). Previous studies have shown that the occurrence of pyroptosis is closely associated with osteoarthritis (157, 158). Within the classical pyroptosis-regulated pathway, the NLRP3 inflammasome activates the protein hydrolase caspase-1, which subsequently regulates the maturation of protein hydrolysis of GSDMD as well as IL-1β and IL-18, and the N-terminus of the hydrolyzed GSDMD pores in the cell membrane, releasing a large amount of inflammatory factors and cellular contents, which ultimately leads to pyroptosis (159). Existing studies support that many risk factors for osteoarthritis, such as obesity, alkaline calcium phosphate and aging, can activate the NLRP3 inflammasome assembly, which induces OA (157, 160, 161). In OA patients, higher levels of pyroptosis-associated inflammatory vesicles and increased levels of the inflammatory factors IL-1β and IL-18 have been detected in the joint fluids, which can increase chondrocyte pyroptosis and inflammation in osteoarthritis (157). Moreover, the inflammatory cytokines IL-1β and IL-18 produced by pyroptosis also play a role in the induction of OA pain and nociceptive sensitization (162). Besides, pyroptosis not only contributes directly to OA, but also interacts with other types of PCD, such as apoptosis, to promote osteoarthritis. Of these, the overproduction of apoptotic vesicles and increased calcification of cartilage tissue can induce cellular pyroptosis, thereby exacerbating osteoarthritis (163). Throughout the development of OA, pyroptosis can cause pathological changes in inflammation-affected joints, such as cartilage defects, cartilage fractures, and arthritis (164).

In several studies, the association of glycolysis-related proteins with pyroptosis has been confirmed. In macrophages, PKM2-dependent glycolysis promotes EIF2AK2 phosphorylation, which activates NLRP3 and AIM2 (165). Evidence suggests that PKM2-mediated upregulation of glycolysis activates NLRP3 inflammasome, leading to pyroptosis in damaged muscle cells (166). Also, the stress granules, acting as cytoplasmic compartments, enable cells to overcome a variety of stressors. For example, the stress granule protein DDX3X interacts with NLRP3 and promotes inflammasome activation, whereas the assembly of stress granules sequesters DDX3X and inhibits NLRP3 inflammasome activation (167). The competition for DDX3X between stress granules and NLRP3 subsequent cell fates under stress conditions determines whether the cell dies. Tetrameric activation of PKM2 ameliorates DDX3X-associated pyroptosis. Thus, prevention of pyroptosis by activation of stress granules through the PKM2 axis is a potential therapeutic approach for inflammatory diseases. Of interest is the fact that inhibition of PFKFB3 could protect intestinal barrier function in sepsis by inhibiting NLRP3/GSDMD (168). ICA has been shown to interfere with the progression of OA by alleviating LPS-induced pyrodeath via inhibiting the NLRP3 inflammator-mediated caspase-1 signaling pathway. It has also been mentioned in related studies that ICA can also increase the expression of some enzymes in the glycolytic pathway, such as phosphoglycerate kinase 1 (PGK1) (94, 169). Several experiments now suggest that inhibition of cellular pyroptosis through glycolytic pathway intervention is some of the therapeutic tools for joint diseases, where TRPV4 mediated mitochondrial dysfunction could induce cellular pyroptosis and cartilage degradation in osteoarthritis through the Drp1- HK2 axis (170). It is also the case that related metabolites in glycolysis, such as lactate, play a critical role in pyroptosis. For example, LDHA-mediated histone lactylation induces cellular pyroptosis by targeting HMGB1 (171). And lactate promotes NLRP3 inflammasome activation, inflammation and cellular pyroptosis, as well as extracellular matrix degeneration in nucleus pulposus cells of degenerating intervertebral discs (172). The regulating of glycolytic homeostasis during OA is a promising strategy to alleviate pyroptosis.

### 4.3 Necroptosis and glycolysis

Necroptosis has been mediated primarily by receptor-interacting protein kinase 1 (RIP1), receptor-interacting protein kinase 3 (RIP3), and mixed-spectrum kinase structural domain-like proteins (MLKLs) (173). The assembly and activation of the RIP1-RIP3 complex are dependent on the kinase activities of both proteins (174). The activation of RIP3 leads to the phosphorylation of MLKL and disruption of the cell membrane (175). The investigation of the role and mechanisms of necrotic apoptosis in OA is a growing area of interest. It now appears that RIP1 expression was found to be significantly increased in cartilage from OA patients and rat models of OA, together with *in vivo* evidence pointing to intra-articular RIP1 overexpression being sufficient to trigger OA symptoms in rats (176–178). These findings emphasise the critical function of RIP1 in the development of OA through the regulation of chondrocyte necrotic apoptosis and ECM catabolism (179). Of interest, it was discovered that bone morphogenetic protein 7 (BMP7) is a novel downstream target of RIP1 in chondrocytes, revealing a non-classical approach to necroptosis regulation (180). The physiological and pathological roles of RIP3 and RIPK1 in the cartilage play a key role and the RIP3-mediated or RIPK1-mediated regulation of necroptosis participates in the etiology of OA (180, 181).

It was found that small colony variants of Staphylococcus aureus compromise host immunity by activating host cell glycolysis to induce necroptosis (182). Acetochlor is known to cause lactate accumulation mediating necroptosis, yet interferes with Warburg-like effects to effectively attenuate necroptosis (183). Hyperglycemia can cause necroptosis of red blood cells and nucleated cells due to glycolysis (184). And the hyperglycemic shift to necroptosis is responsible for events downstream of glycolysis (185). Moreover, it has been suggested that shikonin-induced glycolysis (including the reduction of glucose 6-phosphate and pyruvate and the down-regulation of HK2 and PKM2) can be affected by RIP1 or RIP3 (186). PFKFB3 inhibitors can regulate the interaction of RIP1, RIP3 and MLKL, and induce necrotic apoptosis (187). There is a similar process of glycolysis during osteoarthritis as described above, and therefore intervention in necroptosis can rely on glycolytic interference. However, research in this area is still lacking in OA, and further clinical studies are then needed to directly confirm this intervention process in OA.

### 4.4 Ferroptosis and glycolysis

In 2012, Dixon et al. described for the first time the concept of a novel type of programmed regulated death which was named ferroptosis (188). Ferroptosis was initially thought to be caused by the induction of cell death by the small molecules erastin and RSL-3 in Ras mutant cell lines (189). Iron accumulation and membrane phospholipid peroxidation trigger ferroptosis, a form of cell death caused by damage to cytoplasmic or organelle membranes (190). Ferroptosis showed neither cell swelling in necrosis and cellular burns, nor cell shrinkage and apoptotic vesicle formation in apoptosis. Ferroptosis also exhibited no chromatin condensation or cytoskeletal disintegration in the nucleus. It is however interesting to note that mitochondria in cells undergoing ferroptosis exhibit significant changes including mitochondrial shrinkage, mitochondrial cristae deletion and rupture of the outer mitochondrial membrane (191).

The pathways that trigger ferroptosis are now found to be complex and may be triggered by either extrinsic or intrinsic pathways (192). The extrinsic pathway is generally thought to be initiated by modulation of transporters such as the cystine-glutamate inverse transport system Xc- or activation of the iron transporters transferrin and lactoferrin (193). And it is interesting to note that the intrinsic pathway is mainly induced by blocking the expression or activity of intracellular phospholipid peroxide scavenging systems [e.g., glutathione peroxidase 4 (GPX4)], by excessive phospholipid peroxidation, or by a dysregulation of ferric ion homeostasis (194). Three signature conditions are generally required for the induction of ferroptosis (1): the presence of redox-active iron in the form of unstable iron pools and iron-dependent peroxidases (e.g., lipoxygenase and cytochrome P450) (194) (2); the presence of key substrates for peroxidation, i.e., phospholipids with polyunsaturated fatty acyl termini and diallyl carbons that are susceptible to peroxidation [For example, the classical pathway mediated by ALOXs (195) or POR (196) and the non-classical pathway mediated by the peroxisome-ether-phospholipid axis (197) act on the peroxidation of PUFAs] (3); Dysregulation of complex lipid peroxidation repair networks, including glutathione-GPX4 (188), GCH1-BH4 (198), and NADPH-FSP1-CoQ10 (199–201), leading to disruption of the mitochondrial dihydronicotinic acid dehydrogenase (DHODH) pathway (202). Broadly speaking, ferroptosis is caused by a disruption of the triple dynamic balance between the intracellular ability to scavenge phospholipid peroxidation, represented by GPX4, lipid peroxidation and iron accumulation.

The connections between ferroptosis and OA have been well understood in the recent past. GPX4-dependent chondrocyte ferroptosis has recently attracted more attention in studies related to OA (203, 204).GPX4 can regulate the development of chondrocyte ferroptosis (203, 205). On the other hand, GPX4 can regulate ECM degradation through the MAPK/NF-κB signaling pathway (203). These results convey that a strategy based on GPX4-mediated chondrocyte ferroptosis is a new idea for the treatment of OA. And it is crucial that iron homeostasis is essential for joint health, and joint iron overload has been shown to be closely related to the pathogenesis of OA (206). What’s more, the excess iron ions can induce membrane lipid peroxidation and cellular damage in the articular cells via the Fenton reaction (204, 207). Recent studies have shown that nuclear receptor coactivator 4 (NCOA4) can exacerbate OA by promoting ferritin autophagy-induced chondrocyte ferroptosis (208). What’s more, Xia et al. identified and validated ferroptosis-related genes (ATF3, IL-6, IL-1B, and EGR1) in OA synovial tissues by using bioinformatics analysis, and it is possible that these genes may be associated with synovial hyperplasia (209).

It is relatively easy to associate glycolysis with ferroptosis. PKM2-mediated Warburg effect has been found to cause ferroptosis, and formaldehyde can induce ferroptosis by up-regulating the Warburg effect in hippocampal neuronal cells (210, 211). According to up-regulation analyses of PKM2, formaldehyde increases the Warburg effect in hippocampal tissues, along with lipid ROS levels and iron content. Besides, the suppression of the Warburg effect by dichloroacetic acid (DCA) protected hippocampal neuronal cells from ferroptosis (210). PKM2 deficiency in fibroblasts has been found to impair fibroblast proliferation and promote tubular epithelial cell ferroptosis (212). Also, PKM2-specific loss or modulation of PKM2 activity partially attenuates renal tubular injury and cellular ferroptosis by limiting mitochondrial breaks (213). Alternatively, Apolipoprotein L3 enhances colorectal cancer CD8+ T cell anti-tumor immunity by promoting LDHA-mediated ferroptosis (214). Inhibition of sirtuins6 in pancreatic cancer (PC) can lead to ferroptosis in cells as well as promote LA production and enhance HK2 and LDHA (215). Besides, the LINC02432/Hsa-miR-98–5p/HK2 axis associated with glycolysis regulates the ferroptosis pathway in PC (216). Recombinant pyruvate dehydrogenase kinase isozyme 4 mediated glycolysis in liver fibrosis affects the susceptibility of hepatic stellate cells to ferroptosis (217). Tumor cells have a high rate of glycolysis and at the same time inhibit mitochondrial OXPHOS activity, but when glycolysis is inhibited, this process is reversed and accompanied by increased sensitivity to ferroptosis inducers. The above reversal process may involve several key enzymes of glycolysis, including HK2, PKM2, and platelet-type phosphofructokinase (218). Although there is a similar glycolysis process in OA, the treatment of ferroptosis in OA by glycolysis pathway is still lacking, and it is worth further investigating.

### 4.5 PANoptosis and glycolysis

PANoptosis has recently gained attention as a mode of programmed cell death encompassing the synergistic pathways of apoptosis, necroptosis, and pyroptosis (219). PANoptosis is triggered by certain triggers and controlled by the pan-apoptotic complex (220). Indeed, the extensive association of apoptosis, necroptosis, and pyroptosis appears to be validated by the fact that all three are involved in the process of cellular damage, transformation, and elimination of infected cells (221). PANoptosis has been reported to induce inflammatory cell death by triggering a combination of apoptosis, necroptosis and pyroptosis (222). There are complex activation pathways for PANoptosis. Up to now, several PANoptosome complexes with different sensors and regulators have been identified including ZBP1- (223), AIM2- (224), RIPK1- (225) and NLRP12-PANoptosome (226). It has been shown in a recent bioinformatics study that osteoarthritis is associated with PANoptosis (227). Moreover, three genes, nuclear factor κB inhibitor-α (NFKBIA), ring finger protein 34 (RNF34), and serine dopant 3 (SERINC3), were identified that can be used as characteristic genes of OA and showed good diagnostic predictions for OA. Apart from the previously recounted association of apoptosis, necroptosis and pyroptosis with glycolysis, the association of PANoptosis with glycolysis was also found. It was found that sulconazole induces PANoptosis by triggering oxidative stress and inhibiting glycolysis to increase radiosensitivity in esophageal cancer (228). On the whole, there are still gaps in this area of research, but targeting glycolysis to control PANoptosis in OA is a potential strategy worth looking forward to investigating.

## 5 Necrosis and glycolysis

PCD and necrosis are two very different concepts. Several studies have suggested that necrosis is often considered an alternative to PCD. Necrosis is a form of cell death that does not depend on caspase and energy (229). And it is characterized by 1) irreversible damage to the plasma membrane of the nucleus and destruction of organelles (the process includes karyolysis, pyknosis, and karyorhexis); 2) Cytoplasmic swelling (a process that includes condensation and intense eosinophilia, loss of structure, and fragmentation). Also, the consequences of necrosis affect the groups of cells in the tissue (230).

It was found that necrosis during the immune response may be a normal physiological and procedural event (231). ROS and Ca^2+^ (in ER) affect the process of necrotic cell death (229). Necrotic cell death may increase the release of pro-inflammatory cytokines (e.g., IL-6), thus promoting the inflammatory response (232). Besides, necrosis can not only promote these important pro-inflammatory factors that affect bone damage, but also lead to the formation of osteoclasts and affect the process of bone repair (233). There is evidence for an association between cartilage degeneration and chondrocyte death, which includes the process of necrosis (234). Necrosis has an important association with the pathological mechanism of OA, but further clinical studies are needed to confirm the causal relationship between them. Besides, the relationship between glycolysis and necrosis remains unclear. Therefore, targeting glycolysis to control necrosis in OA patients is a potential research gap worth exploring.

## 6 Diagnosis and treatment of glycolysis in OA

The potential diagnostic and therapeutic value of the aerobic glycolytic pathway in OA has been confirmed by several studies. Many of the enzymes involved in the aerobic glycolysis of OA can be used as biomarkers for the diagnosis of OA. For example, the increased expression of HK2 can be found in peripheral blood monocytes of patients with OA, which has a certain reference value for clinical diagnosis (235). In synovial fluid of OA patients, LDHA activity levels and lactate concentrations are significantly higher than in normal synovial fluid, according to Li et al. (24). Several studies have identified PFKB3 in aerobic glycolysis as a valuable biomarker for identifying patients with OA (109). In addition, a proportional correlation between aerobic glycolysis and the levels of some proinflammatory immune cells (e.g., M1 and M2 macrophages) in skeletal joints may be used to determine the development of OA (122).

In terms of treatment, there are also many related studies proposing to improve the condition and protect cartilage by targeting the glycolytic process in OA. For example, the link between PKM2 and OA has been further demonstrated by studies showing that possible ways to alleviate synovial inflammation and clinical symptoms associated with OA are PKM2 isoform degradation in macrophages was enhanced by low-intensity pulsed ultrasound to inhibit the production of mature IL-1β and autophagy-mediated sequestosome 1 (SQSTM1) was enhanced to macrophages treated with LPS-adenosine triphosphate (ATP), and this study provides new ideas for non-invasive treatment of OA (36). Besides, GSK2837808A (GSK, an LDHA blocker) has been found in a study investigating the relationship between LDHA and OA in order to provide an effective treatment that can reverse the pathological state of OA by reducing the inflammatory response (i.e., expression of the inflammatory cytokine IL-1β), the level of lactate production, the uptake of glucose (without affecting normal ATP production), and the production of adenosine monophosphate (AMP) in OA (24). Besides, extracts of some Chinese medicinal herbs, such as icariin, achyranthes bidentata extracts, and andrographolide, can reduce synovial inflammation, inhibit chondrocyte apoptosis and improve joint hypoxia environment in OA by regulating several important enzymes (e.g., PKM2 and HK2) and processes in aerobic glycolysis through some signaling pathways (e.g., PI3K/AKT, MAPK, and NF-κB/HIF-2α) (95, 122). We have summarized the related parts in **Table 1**. However, the protective and therapeutic mechanism of aerobic glycolysis inhibition in OA has not been fully elucidated, and the specific mechanism of related enzyme inhibitors and drugs and the possible side effects or adverse reactions need to be further studied to prove.

**Table 1:** Treatment of OA by aerobic glycolysis.

Drug	Target	Effect	Reference
**GSK2837808A**	LDHA	• Imped lactate secretion • Promote HAS2 and hyaluronic acid synthesis	(100)
**FX11**	LDHA	• Decrease ROS	(22)
**Anti-PKM2**	PKM2	• Mitigated ER stress‐mediated apoptosis process and subsequent inflammatory injury triggered by IL‐1β in rat chondrocytes	(24)
**cDNA oligonucleotides**	PKM2	• Suppressed OA chondrocyte proliferation and promote apoptosis • Decrease collagen matrix generation in OA chondrocyte • Decrease the energy accommodation and then attenuate extracellular collagen matrix generation in OA chondrocyte	(38)
**Achyranthes bidentata extract**	glycolysis	• Facilitate the cell proliferation • Suppress chondrocytes apoptosis and glycolytic activity	(71)
**Baricitinib or tofacitinib;** **3-BrP and FX11**	Janus kinases and glycolytic enzymes (including HKII, PFKp, PKM2, LDH-A)	• Reduce lactate production • Downregulate the secretion of IL-6 and MMP3	(12)

Programmed cell death protein-1(PD-1), Dichloroacetic acid (DCA), 2-deoxy-d-glucose (2-DG), and 3-bromopyruvate (3-BrpA) are used as therapeutic agents and drugs in cancer, hereditary mitochondrial diseases and some chronic diseases in clinical (236–238). It has been suggested that the deficiency of PD-1 triggers the occurrence of aerobic glycolysis, which facilitates the fermentation of glucose into LA (237). DCA, 3-BrpA and 2-DG act as glycolytic inhibitors by promoting the oxidative capacity of glucose during glycolysis and inhibiting key enzymes (e.g., HK2) during aerobic glycolysis (236, 238). Besides, the clinical treatment of some diseases (e.g., cancer) by regulating the process of glycolysis has also been demonstrated in the Complementary and alternative medicine (CAM) of Apiumgraveolensvar.dulce (as HIF-1α inhibitor), Spatholobus suberectus (as an LDH-A inhibitor) and the Ben Cao Xiao Ke Dan (as a HK2 inhibitor) (239). However, clinical evidence for the use of these drugs in OA is still lacking, so further clinical studies are still needed to investigate.

## 7 Conclusion

In recent years, with the rise of studies in the new field of aerobic glycolysis as a pathological mechanism of OA, its potential as a targeted therapy for cartilage injury has attracted more and more attention. Apoptosis and inflammation have always been the main focus of the treatment of OA. With the continuous innovation of research perspectives, the relationship between the key enzymes involved in aerobic glycolysis and the regulation of the pathological mechanism of OA has been further established.

However, there are still many problems in upregulating the expression of glycolytic enzymes in osteoarthritic cartilage with drugs, and more precise targeted therapies need to be found from the perspective of biochemistry and clinical medicine. The effects produced by interfering with glycolysis in OA still warrant more in-depth investigation, as is the case in cancer therapy, where inhibition of glycolysis can reduce tumor progression while causing a variety of side effects. For example, undirected inhibition of PKM2 does reduce tumor progression, but PKM2 dominates metabolism in specific cells (e.g., endothelial cells), so that the inhibition of PKM2 results in a breakdown of vascular endothelial homeostasis. It has not been fully elucidated the exact roles and mechanisms of action of glycolytic enzymes such as PKM2 in different cells. In addition, broad-spectrum inhibition of glycolysis and its major enzymes may be a double-edged sword in the treatment of OA. Shi et al. have presented a very worthwhile point of view that needs attention (240). To prevent OA without harming PKM2-dependent cells, studies should explore targeting PKM2 specifically.

So on, the use of some chemical inhibitors by interfering with glycolysis to treat OA in clinical application is still in doubt, because the treatment regulation of key enzymes (e.g., PKM2, HK2, and LDHA, etc.) is the basis of the cell respiration substrates, so how to select the precise drug delivery method to target to OA of aerobic glycolysis process is very important. With the advancement of medical technology, some biomaterials have entered the medical field of view, which can be used to deliver cells. Hydrogels, for example, are chemically cross-linked transport vehicles that regulate cells to reach their target sites and can be used clinically by injection or minimally invasive means. Some hydrogels (e.g., alginate saline gels) have been used for cartilage regeneration and cartilage formation. Therefore, how to target the key enzymes of glycolysis by changing the mechanical properties of hydrogels is a direction that can be further investigated (241). In addition to hydrogels, sponges, foams and fibrous webs also have related applications in cartilage regeneration. However, the use of these biomaterials still needs a lot of research to improve the synthesis of their structural and functional properties. Moreover, stem cell therapy for OA is gradually entering the field of clinical research. It has been proved that the use of stem cell therapy to inject collagenase into the joint can achieve the therapeutic purpose of cartilage protection and reduce the clinical symptoms of OA (242, 243). Therefore, the extraction and application of glycolytic key enzymes have the potential to be used in the minimally invasive treatment of OA. Currently, drugs targeting aerobic glycolysis in OA cannot be used as a therapeutic approach to the clinic, because the current research is limited to animal models and *in vitro* cell tests. Thus, while using different types of carrier delivery intervention drugs related to OA of aerobic glycolysis is an effective method for the potential is considerable, but still need to be before entering into clinical research concern on drug use (e.g., the stability of the drug carrier with combining, and drug leakage outside the carrier into the articular cartilage may cause potential adverse reactions). On the other hand, it is gratifying to note that several foods and ethnomedicines (e.g., TCM) have the potential to rescue OA by their unique multi-targeted and low-toxicity effects on glycolysis-related pathways. These findings could offer much promise for therapeutic advances in OA.

In conclusion, this review has elucidated many biological mechanisms and signaling pathways involved in the regulation of OA by aerobic glycolysis, and the intervention of aerobic glycolysis to protect cartilage and reduce cartilage degradation is the key point for the reversal of OA chondrocyte activity. It has shown that glycolysis is closely related to the related pathological mechanisms such as the elimination of inflammation and the reduction of apoptosis in OA treatment. Therefore, the rational targeting of enzymes or intervention with a certain process of glycolysis during aerobic glycolysis is a promising option for the development of non-surgical treatment of OA in the future biomedical field.

## Author contributions

DJ: Data curation, Investigation, Methodology, Writing – original draft. JG: Data curation, Investigation, Methodology, Writing – original draft. YL: Data curation, Investigation, Methodology, Writing – original draft. WL: Data curation, Investigation, Writing – original draft. DL: Conceptualization, Funding acquisition, Project administration, Supervision, Writing – review & editing.
